# Dynamic Change in Insulin Resistance Induced by Free Fatty Acids Is Unchanged Though Insulin Sensitivity Improves Following Endurance Exercise in PCOS

**DOI:** 10.3389/fendo.2018.00592

**Published:** 2018-10-05

**Authors:** Myint Myint Aye, Alexandra E. Butler, Eric S. Kilpatrick, Richard Kirk, Rebecca Vince, Alan S. Rigby, Derek Sandeman, Stephen L. Atkin

**Affiliations:** ^1^Department of Academic Endocrinology, Diabetes and Metabolism, Hull York Medical School, Hull, United Kingdom; ^2^Diabetes Research Centre, Qatar Biomedical Research Institute, Doha, Qatar; ^3^Sidra Medical Research Centre, Doha, Qatar; ^4^Department of Sport, Health and Exercise Science, Hull York Medical School, The University of Hull, Hull, United Kingdom; ^5^The University of Hull, Hull York Medical School, Hull, United Kingdom; ^6^Department of Diabetes and Endocrinology, University Hospital Southampton NHS Foundation Trust, Southampton, United Kingdom; ^7^Weill Cornell Medicine Qatar, Education City, Doha, Qatar

**Keywords:** insulin resistance, intralipid, endurance exercise, PCOS, insulin sensitivity

## Abstract

**Background:** Insulin resistance (IR) is the hallmark of PCOS and it is known that exercise may decrease it. What is unknown is whether exercise may mechanistically alter the underlying IR, attenuating the dynamic lipid induced IR in insulin resistant subjects.

**Methods:** 12 women with polycystic ovary syndrome (PCOS) and 10 age and body mass index matched controls completed an 8 week supervised exercise program at 60% maximal oxygen consumption. Before and after the exercise program, all participants underwent hyperinsulinaemic euglycaemic clamps with either saline or intralipid infusions. Skewed data were log transformed and expressed as mean ± SEM.

**Results:** Before exercise, women with PCOS had a higher HOMA-IR and lower VO_2_ max than controls. Compared to saline, lipid infusion lowered the rate of insulin stimulated glucose disposal (M value; mg/kg/min) by 67 ± 5% (from 0.5 ± 0.03 to −0.25 ± 0.2, *p* = 0.01) in PCOS, and by 49 ± 7% (from 0.65 ± 0.06 to 0.3 ± 0.1, *p* = 0.01) in controls. The M value was significantly less in PCOS compared to controls for both saline (*p* < 0.01) and lipid (*p* < 0.05). Endurance exercise in PCOS improved VO_2_ max and HOMA-IR, but not weight, to those of pre-exercise control subjects. The glucose disposal rate during the lipid infusion was reduced following exercise in PCOS, indicating decreased IR (67 ± 5 vs. 50 ± 7%, *p* = 0.02), but IR was not altered in controls (49 ± 7 vs. 45 ± 6%, *p* = 0.58). The incrementally increased IR induced by the lipid infusion did not differ between controls and PCOS.

**Conclusion:** Insulin sensitivity improved with exercise in the PCOS group alone showing that IR can be modified, though likely transiently. However, the maximal IR response to the lipid infusion did not differ within and between control and PCOS subjects, indicating that the fundamental mechanism underlying insulin resistance was unchanged with exercise.

**Precis:** Maximal insulin resistance induced by lipid infusion determined at baseline and 8 weeks after exercise in control and PCOS women did not differ, though insulin sensitivity increased in PCOS after exercise.

## Introduction

Non-esterified fatty acids (NEFA) play a significant role in the pathophysiology of insulin resistance (1). Increased availability of NEFA could lead to storage of lipids “ectopically” in tissues other than adipocytes, such as muscle, liver and beta cells (2). The intramuscular triglyceride (IMTG) storage is positively correlated with the degree of total body and visceral fat (3, 4). Increased availability coupled with a decreased rate of mitochondrial lipid oxidation in skeletal muscle results in accumulation of intra-myocellular lipid metabolites such as the long chain fatty acids Acyl Co A, diacylglycerol (DAG) and ceramides (5, 6). These are thought to interfere with insulin signaling mediated glucose transport, and thus result in insulin resistance (IR) in skeletal muscle (7, 8). An acute fat load orally or intravenously results in an acute rise in DAG in the muscle that subsequently induces insulin resistance by reducing nonoxidative glucose disposal through PKCθ activation (9). Furthermore, an acute rise in NEFA levels decreases insulin stimulated glucose disposal in skeletal muscle and increases hepatic glucose production, resulting in IR (10).

Fatty acids are the predominant fuel used by skeletal muscle during fasting and exercise. Exercise increases the blood flow through the adipose tissue and enhances the delivery of free fatty acids for use by skeletal muscle. Exercise improves fatty acid oxidation and reduces accumulation of intra-myocellular fat metabolites and subsequently improves IR (11). A maximal rate of fat oxidation is observed at exercise intensities between 59 and 64% of maximum oxygen consumption (VO_2_ max) in trained individuals, and between 47 and 52% of that in a large sample of the general population (12). Bruce et al. illustrated that moderate intensity exercise increases mitochondrial fatty acid oxidation and decreases DAG and ceramide content of skeletal muscle in obese subjects (11). Schenk et al. demonstrated that one session of exercise completely decreased the accumulation of highly bioactive fatty acid metabolites, and then reversed fatty acid-induced IR, in healthy subjects (13).

Polycystic ovary syndrome (PCOS) is a dysmetabolic condition that is strongly associated with obesity, IR and metabolic dyslipidaemia. Women with polycystic ovary syndrome (PCOS) showed lower insulin sensitivity (IS) than women without PCOS, and the impact of BMI was greater on IS in women with PCOS than those without PCOS (14). In addition, PCOS that were overweight showed moderately lower IS than lean PCOS (15). The prevalence of gestational diabetes, impaired glucose tolerance and type 2 diabetes (5-fold in Asia, 4-fold in the Americas and 3-fold in Europe) are significantly increased in PCOS regardless of their age, with risk independent of, yet exacerbated by, obesity (16).

PCOS is well recognized to have an intrinsic post-receptor insulin signaling defect in skeletal muscle (17, 18) that may augment the detrimental effect of NEFA on insulin sensitivity. Therefore, raised NEFA levels may worsen IR to a greater extent in women with PCOS than those without PCOS, but it is unknown if this mechanism can be modified. A lipid infusion gives a dynamic and maximal measure of IR, and the gold standard to measure IR is the hyperglycemic glucose clamp method to determine its change with an intervention. The effect of endurance exercise on lipid induced IR in subjects with underlying IR has not been studied, nor is it known if the underlying mechanism of IR is altered by exercise. We therefore hypothesized that women with PCOS were less tolerant to an acute rise in NEFA levels resulting in more severe IR than weight and age matched healthy subjects, and that endurance exercise would improve their tolerance to lipid induced IR with a mechanistic change in IR.

## Subjects and methods (figure 1)

This study was approved by a local research ethics committee. Subjects with PCOS were recruited from the endocrine clinics, and normal female controls were recruited through advertisements at a local university and in the hospital newsletters. PCOS was diagnosed based on the presence of two out of three criteria; oligomenorrhoea, clinical or biochemical hyperandrogenism, and polycystic ovaries on ultrasound after exclusion of other endocrine causes of hyperandrogenism according to the Rotterdam criteria (19); however all women fulfilled all 3 criteria. All the subjects gave their informed consent to participate in the study. They were all non-smokers, took no regular medications, had no concurrent illness and had had no regular exercise prior to the study. All women had a pregnancy test prior to their inclusion in the study. Subjects who were diagnosed with impaired glucose tolerance at screening oral glucose tolerance test were excluded. Subjects were requested not to modify other aspects of their lifestyle, including their dietary pattern, during the study period. All the subjects had anthropometric measurements, fasting blood sampling and two hyperinsulinaemic euglycaemic clamps with either 5 h saline or intralipid infusions at baseline. Normal control women had the initial clamp in the first week of their menstrual cycle, whilst PCOS women were clamped after 6 weeks amenorrhea. These tests were repeated after completion of an 8 week moderate intensity exercise program.

**Figure 1:**
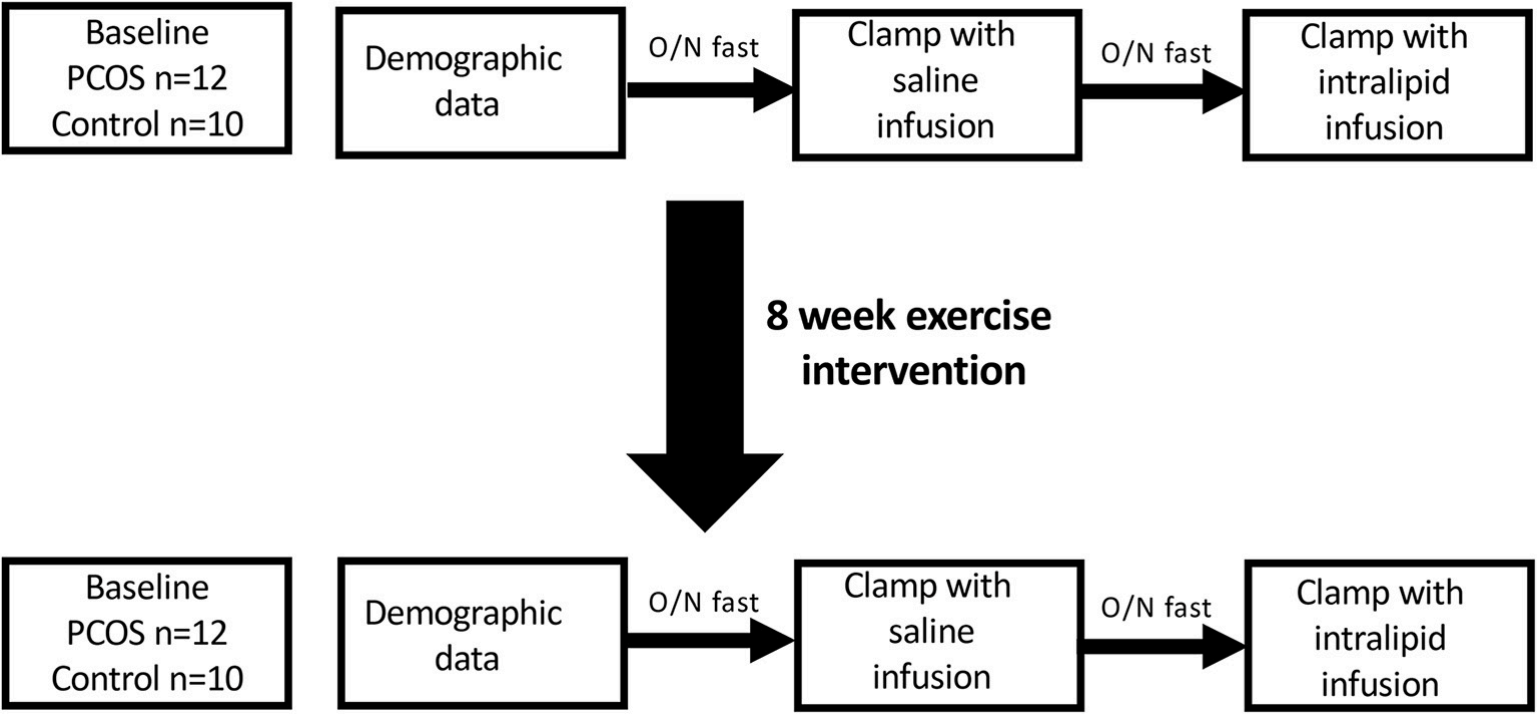
Overall study scheme.

### Hyperinsulinaemic euglycaemic clamps

Following a 12 h overnight fast, participants underwent a hyperinsulinaemic euglycaemic clamp that measured insulin sensitivity whilst receiving an infusion of normal saline on the first occasion followed by a second intralipid infusion within a week. An 18 gauge intravenous cannula was inserted into an antecubital vein to administer test infusions and a retrograde cannula was inserted into the dorsal hand vein on the contralateral hand. This hand was heated (60°C) to arterialize venous blood for the measurement of blood glucose. On the lipid infusion day, an additional cannula was placed in the contralateral antecubital vein for the lipid emulsion. After fasted blood samples were taken, either normal saline 1.5 mL/min or 20% intralipid 1.5 mL/min along with unfractionated heparin sodium 0.3 unit/kg/min was infused for 5 h. At 180 min, a 2 h hyperinsulinaemic euglycaemic clamp was started using intravenous soluble insulin (Humulin S, Eli Lilly and Co., Indianapolis, IN) at a rate of 80 mU/m^2^ surface area/minute for the first 20 min followed by a constant rate of 40 mU/m^2^ surface area/minute for the remaining 100 min. Plasma glucose was clamped at 5.0 mmol/L with a variable infusion rate of 20% dextrose, adjusted relative to arterialized blood glucose measurements undertaken every 5 min. Endogenous glucose production was more than 90% suppressed by an acute rise of insulin with the primed insulin infusion (20). The rate of insulin stimulated glucose disposal (mg/kg/min) (M value), a measure of insulin sensitivity, was calculated from the mean of the five 20 min periods from 20 to 120 min during the clamp using the Defronzo method (21). Blood samples were taken at baseline and every hour for 5 h. These samples were centrifuged at 1,500 G for 15 min at 4°C within 15 min of sampling, and then plasma and serum stored at −80°C until analysis.

### Exercise intervention

All participants underwent a structured supervised exercise program for a 1 h session, three times per week for 8 weeks in the sports laboratory at the Department of Sports, Health and Exercise Science, the University of Hull. Moderate intensity exercise was defined as achieving a targeted heart rate equivalent to 60% of their baseline VO_2_ max. Measurement of individualized VO_2_ max was performed on a motorized treadmill starting with a warm up, then speeding to walking pace then increasing every minute until the subject could not keep pace with the treadmill or became too tired to continue. Inspired and expired gas fractions, and heart rate were continuously monitored (22). All supervised exercise sessions achieved a targeted heart rate equivalent to 60% of their baseline VO_2_ max. A mid-point reassessment was conducted to adjust the moderate intensity based on improvements in exercise capacity.

### Biochemical analysis

Serum insulin was assayed using a competitive chemiluminescent immunoassay (Euro/DPC, Llanberis, UK). Plasma glucose was measured using a Synchon LX 20 analyzer (Beckman-Coulter, High Wycombe, UK). Serum testosterone was measured by high performance liquid chromatography linked to tandem mass spectrometry (Waters Corporation, Manchester, UK) and sex hormone binding globulin (SHBG) was measured by immunometric assay with fluorescence detection on the DPC Immulite 2000 analyzer. The free androgen index (FAI) was obtained as the quotient 100^*^ Testosterone/SHBG. Total cholesterol, triglycerides (TG), and high density lipoprotein cholesterol (HDL-c) were measured enzymatically using a Synchon LX20 analyzer (Beckman-Coulter, High Wycombe, UK). LDL-c was calculated using the Friedewald equation (23). Non-esterified fatty acids (NEFA) were analyzed using enzymatic colorimetric methods (Wako NEFA-H2) on a Konelab20 autoanalyzer with an inter-assay and the coefficient of variation was 1.4%.

### Data analysis

Homeostatic model assessment of insulin resistance (HOMA-IR) was calculated by the formula: HOMA1-IR = fasting plasma insulin (μU/ml) × fasting plasma glucose (mmol/L)/22.5 (24). Total TG are under the response curve (AUC), and insulin, glucose and NEFA AUC were calculated using a trapezoidal rule (25).

Statistical analysis was performed using SPSS for Windows NT, version 19.0 (SPSS Inc., Chicago, IL). Wilcoxon signed ranks test was applied to skewed variables that violated the assumptions of normality when tested with the Kolmogorov–Smirnov test, and paired sample *t* test for normally distributed data within the group. Log transformation and then an unpaired *t* test were undertaken on those data that were skewed. Data are presented means ±SEMs. For all analyses, a two-tailed *P* ≤ 0.05 was considered to indicate statistical significance. The missing values were handled by case-wise deletion.

## Results

Twelve women with PCOS and ten healthy subjects, comparable in terms of age and BMI, completed the study. Baseline characteristics of the subjects are summarized in Table 1. Subjects with PCOS had a larger waist hip ratio, higher FAI and HOMA-IR, and lower HDL-c than controls. Fasting TG and NEFA were not increased in PCOS relative to controls. PCOS subjects were less physically fit, with a lower VO_2_ max than controls.

**Table 1:** Baseline characteristics of participants.

	**Controls (*n* = 10)**	**PCOS (*n* = 12)**
Age (year)	25.26 ± 6.5	28.27 ± 6.5
BMI (kg/m2)	26.8 ± 6.5	29.4 ± 5.5
Waist (cm)	81.1 ± 14.2	98.8 ± 15.8
WHR	0.79 ± 0.06	0.87 ± 0.06
Testosterone (nmol/L)	1.05 ± 0.34	1.51 ± 0.69
FAI	2.1 ± 2.05	6.77 ± 2.90
SHBG (nmol/L)	69.6 ± 29.7	26 ± 17.6
TC (mmol/L)	4.61 ± 0.75	4.13 ± 0.65
TG (mmol/L)	0.84 ± 0.18	1.25 ± 0.72
HDL-c (mmol/L)	1.48 ± 0.47	1.12 ± 0.20
LDL-c (mmol/L)	2.66 ± 0.56	2.33 ± 0.51
FPG (mmol/L)	4.89 ± 0.56	4.94 ± 0.55
HbA1c (mmol/mmoL)	33 ± 5.6	34 ± 2.9
TSH (iu/L)	1.9 ± 0.91	1.6 ± 0.58
HOMA-IR	1.34 (0.80, 2.13)	2.3 (1.3, 3.9)
NEFA (mmol/L)	0.48 (0.29, 0.64)	0.45 (0.42, 0.58)
VO2 max (ml/kg/min)	36.3 ± 6.3	26.9 ± 4.8

*Skewed variables are provided as the medians (25th, 75th percentile). Normally distributed variables are shown as the means ± standard deviation*.

### Effect of lipid on insulin resistance before exercise intervention (tables 2, 3)

During the saline infusion, PCOS subjects had a lower rate of insulin stimulated glucose disposal (0.65 ± 0.06 vs. 0.5 ± 0.03 mg/kg/min, *p* = 0.01) but higher TG AUC 3 h (3.51 ± 0.56 vs. 2.34 ± 0.14) mmol/L, *p* = 0.05 than controls. Lipid infusion led to a 3.5–4 fold rise in NEFA AUC and TG AUC in both groups. This led to a reduction in insulin stimulated glucose disposal rate in PCOS from 0.5 ± 0.03 to −0.16 ± 0.2) mg/kg/min, *p* = 0.01 and in controls from 50.65 ± 0.06 to 0.3 ± 0.1 mg/kg/min, *p* = 0.01. The glucose disposal rate fell more in PCOS than in controls from their (saline) baseline levels following lipid infusions (67 ± 5 vs. 49 ± 7%, PCOS vs. control, *p* = 0.05). Therefore, PCOS subjects were more susceptible to lipid induced insulin resistance. In addition, the lipid infusion caused a rise in plasma glucose AUC 3 h in PCOS from 14.2 ± 0.26 to 14.9 ± 0.25 mmol/L, *p* = 0.03 but with no concomitant increase in insulin secretion [insulin AUC 3 h (pmol/L): with saline 2.1 ± 0.0 with intralipid 2.2 ± 0.08]. The within group effect of the intralipid infusion on insulin sensitivity is summarized in Table 2.

**Table 2:** Effect of intralipid infusion on insulin sensitivity within groups.

	**Controls**	**PCOS**	**PCOS-Controls**	
	**Mean (After-before)**	**Mean (After-before)**	**Mean difference (95% CI)**	***p*-value**
Glucose disposal mg/kg/min	−2.518	−2.076	0.51 (0.49, 0.54)	0.33
NEFA AUC 3 h (mmol/L)	4625	5922	1558 (1496, 1620)	0.19
TG AUC 3 h (mmol/L)	8.24	9.01	0.60 (0.48, 0.72)	0.63
Glucose AUC 3 h (mmol/L)	0.65	0.73	−0.058 (−0.092, 0.024)	0.88
Insulin AUC 3 h (pmol/L)	−16.64	38.23	57.58 (55.66, 59.50)	0.49
NEFA AUC 5 h (mmol/L)	9703	11137	1987 (5572, 5782)	0.36
TG AUC 5 h (mmol/L)	18.2	18.4	−0.54 (−14.77, 13.69)	0.94

**Table 3:** Effect of intralipid infusion on insulin sensitivity between groups.

	**Controls**	**PCOS**	**PCOS-Controls**	
	**Mean (After-before)**	**Mean (After-before)**	**Mean difference (95% CI)**	***p*-value**
Glucose disposal mg/kg/min	−2.518	−2.076	0.512 (0.489, 0.541)	0.33
NEFA AUC 3 h (mmol/L)	4625	5922	1558 (1496, 1620)	0.19
TG AUC 3 h (mmol/L)	8.24	9.01	0.60 (0.48, 0.72)	0.63
Glucose AUC 3 h (mmol/L)	0.65	0.73	−0.058 (−0.092, 0.024)	0.88
Insulin AUC 3 h (pmol/L)	−16.64	38.23	57.58 (55.66, 59.50)	0.49
NEFA AUC 5 h (mmol/L)	9703	11137	1987 (5572, 5782)	0.36
TG AUC 5 h (mmol/L)	18.2	18.4	−0.54(−14.77, 13.69)	0.94

When the effect of the intralipid infusion on insulin sensitivity between the PCOS and control groups was analyzed, no differences were apparent (Table 3).

### Effect of exercise on demographic and fasting biochemical parameters (tables 4, 5)

The endurance exercise for 8 weeks improved cardiovascular fitness in both PCOS patients and controls (mean VO_2_ max ml/kg/min ± SEM: PCOS before 8 weeks exercise, 26.9 ± 1.40; PCOS after 8 weeks exercise, 28.7 ± 1.7, *p* = 0.05) and (mean VO_2_ max ml/kg/min± SEM: controls before 8 week exercise, 36.3 ± 2.02; controls after 8 week exercise, 39.2 ± 1.8; *p* = 0.008). This was accompanied by a significant reduction in HOMA-IR in PCOS from 1.2 ± 0.09 to 1.07 *p* = 0.01) although this did not reach significance in controls (from 0.94 ± 0.11 to 0.86 ± 0.1; *p* = 0.08). The post exercise HOMA-IR level of PCOS was reduced to the pre-exercise level of controls. The reduction in IR was associated with a reduction in waist circumference (98.1 ± 4.6 vs. 96.4 ± 4.4 cm, *p* = 0.05) but no significant changes in weight (29.4 ± 1.6 vs. 29.1 ± 1.8 kg/m2, *p* = 0.40) in PCOS. Total cholesterol, LDL-c, HDL-c and NEFA levels were not decreased post-exercise, but TG fell in both groups (Table 4).

**Table 4:** Demographic and biochemical changes with exercise within groups.

**Parameters**	**Controls (*****n*** = **10)**			**PCOS (*****n*** = **12)**		
	**Exercise**			**Exercise**		
	**Before**	**After**	***p* =**	**Before**	**After**	***p* =**
BMI (kg/m2)	26.8 ± 2.0	26.5 ± 1.5	0.07	29.4 ± 1.6	29.1 ± 1.8	0.40
Waist (cm)	81.1 ± 4.5	80.1 ± 3.6	0.20	98.1 ± 4.6	96.4 ± 4.4	0.05
TC (mmol/L)	4.61 ± 0.24	4.44 ± 0.27	0.29	4.13 ± 0.19	3.95 ± 0.24	0.26
TG (mmol/L)	0.84 ± 0.06	0.69 ± 0.07	0.03	1.25 ± 0.21	0.97 ± 0.12	0.06
HDL-c (mmol/L)	1.48 ± 0.15	1.6 ± 0.12	0.29	1.12 ± 0.06	1.13 ± 0.06	0.78
LDL-c (mmol/L)	2.66 ± 0.18	2.68 ± 0.23	0.99	2.33 ± 0.15	2.12 ± 0.14	0.11
^*^NEFA (mmol/L)	2.65 ± 0.05	2.66 ± 0.06	0.89	2.65 ± 0.04	2.65 ± 0.02	0.86
FPG (mmol/L)	4.89 ± 0.18	4.74 ± 0.15	0.26	4.94 ± 0.16	431 ± 0.40	0.18
^*^HOMA-IR	0.94 ± 0.11	0.86 ± 0.10	0.08	1.2 ± 0.09	1.07 ± 0.08	0.01
VO_2_ max (ml/kg/min)	36.3 ± 2.02	39.2 ± 1.8	0.008	26.9 ± 1.40	28.7 ± 1.7	0.05

* *Log transformed t test (±SEM)*.

**Table 5:** Demographic and biochemical changes with exercise between groups.

	**Controls**	**PCOS**	**PCOS-Controls**	
**Exercise**	**Mean (After-before)**	**Mean (After-before)**	**Mean difference (95% CI)**	***p*-value**
BMI (kg/m2)	−0.18	−0.16	0.06 (0.02, 0.10)	0.97
Waist (cm)	−2.1	−2.2	−0.10 (−0.22, 0.022)	0.96
TC (mmol/L)	−0.17	−0.2	−0.03 (−0.05, 0.01)	0.91
TG (mmol/L)	−0.19	−0.01	0.18 (0.16, 0.37)	0.16
HDL-c (mmol/L)	−0.07	−0.02	0.05 (0.04, 0.06)	0.54
LDL–c (mmol/L)	0	−0.28	−0.28 (−0.29, −0.27)	0.17
NEFA (mmol/L)	0.23	−37.44	−37.67 (−44.86, −30.48)	0.70
FPG (mmol/L)	−0.04	−0.27	−0.23 (−0.26, −0.20)	0.66
HOMA–IR	0.58	−0.25	-0.83 (−0.86, −0.80)	0.16
VO_2_ max (ml/kg/min)	8.05	−4.89	−12.94 (−13.25, −12.63)	< 0.005

There were no significant differences between groups in demographic or biochemical data following exercise, with the exception of with VO_2_ max, where the control group showed a significant improvement with exercise while the PCOS group did not (Table 5).

### Effect of exercise on lipid induced insulin resistance (figure 2, tables 6, 7)

**Figure 2:**
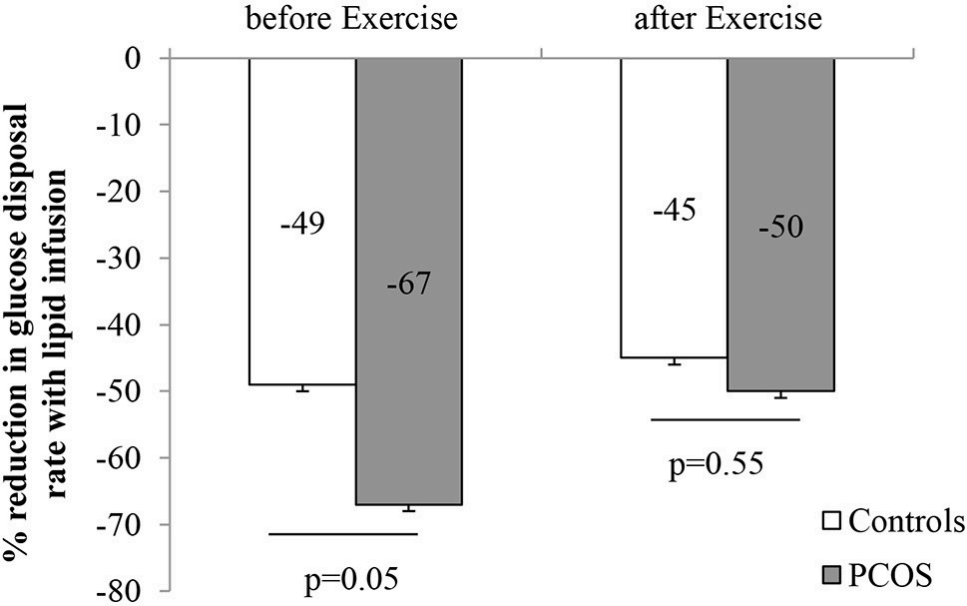
Effect of intralipid on the rate of glucose disposal before and after moderate intensity exercise (Data are expressed as mean ± SEM).

After 8 weeks of moderate intensity exercise, there were concomitant rises in insulin sensitivity, in both PCOS patients and controls (Table 6). More importantly, exercise improved lipid induced insulin resistance in both group (Table 6). In PCOS, endurance exercise improved lipid induced insulin resistance i.e. lipid infusion reduced glucose disposal rate by 67% before exercise and 50% after exercise (*p* = 0.02). The effect of lipid infusion on glucose disposal rate between controls (before exercise) and PCOS (after exercise) was similar (49 ± 7 vs. 50 ± 7%, *p* = NS) (Figure 2). Exercise decreased TG AUC in PCOS (*p* < 0.05), and lowered the 3 h AUC NEFA levels significantly (Table 6).

**Table 6:** Effect of exercise on intralipid-induced insulin resistance within groups.

		**Controls (*****n*** = **10)**			**PCOS (*****n*** = **12)**		
		**Exercise**			**Exercise**		
**Parameter**	**Infusion**	**Before**	**After**	***p* =**	**Before**	**After**	***p* =**
^*^Glucose disposal mg/kg/min	Saline	0.65 ± 0.06	0.74 ± 0.05	0.01	0.5 ± 0.03	−0.25 ± 0.03	0.01
	Intralipid	0.30 ± 0.10	0.42 ± 0.08	0.01	−0.16 ± 0.2	−0.79 ± 0.17	0.01
Lipid induced % reduction in glucose disposal rate		49 ± 7%	45 ± 6%	0.58	67 ± 5%	50 ± 7%	0.02
^*^NEFA AUC 3 h (mmol/L)	Saline	2.9 ± 0.05	2.5 ± 0.10	0.002	3.0 ± 0.02	2.7 ± 0.05	0.003
	Intralipid	3.0 ± 0.09	3.0 ± 0.11	0.60	2.7 ± 0.07	2.3 ± 0.02	0.0001
TG AUC 3 h (mmol/L)	Saline	2.34 ± 0.14	2.51 ± 0.41	0.70	3.51 ± 0.56	2.83 ± 0.48	0.05
	Intralipid	11.0 ± 1.28	9.35 ± 0.78	0.09	13.4 ± 1.61	10.7 ± 1.44	0.03
Glucose AUC 3 h (mmol/L)	Saline	14.4 ± 0.40	14.1 ± 0.26	0.38	14.2 ± 0.26	14.01 ± 0.49	0.65
	Intralipid	15.0 ± 0.24	14.9 ± 0.24	0.86	14.9 ± 0.25	14.4 ± 0.28	0.17
^*^Insulin AUC 3 h (pmol/L)	Saline	2.0 ± 0.08	2.0 ± 0.07	0.87	2.1 ± 0.07	2.2 ± 0.08	0.56
	Intralipid	1.9 ± 0.08	2.0 ± 0.09	0.91	2.2 ± 0.09	2.2 ± 0.08	0.83

*Normally distributed variables are shown as mean ± SEM*.

* *Log transformed t test (±SEM)*.

**Table 7:** Effect of exercise on intralipid-induced insulin resistance between groups.

		**Controls**	**PCOS**	**PCOS-Controls**	
**Exercise**	**Infusion**	**Mean (After-before)**	**Mean (After-before)**	**Mean difference (95% CI)**	***p*-value**
Glucose disposal mg/kg/min	Saline	0.56	0.72	0.16 (0.12, 0.19)	0.8
	Intralipid	0.56	0.28	−0.28 (−0.30, −0.26)	0.41
NEFA AUC 3h (mmol/L)	Saline	−405.94	−244.41	161.52 (146.11, 176.94)	0.5
	Intralipid	−606.07	−1129.59	−523.52 (−594.28, −452.76)	0.64
TG AUC 3h (mmol/L)	Saline	0.17	−0.78	0.86 (0.82, 0.91)	0.13
	Intralipid	−1.64	−2.32	−2.49 (−2.56, −2.41)	0.63
Glucose AUC 3h (mmol/L)	Saline	−0.36	−0.15	0.21 (0.18, 0.25)	0.69
	Intralipid	−0.08	−0.34	−0.26 (−0.30, −0.22)	0.64
Insulin AUC 3h (pmol/L)	Saline	−2.14	1.70	3.84 (1.87, 5.81)	0.91
	Intralipid	−0.76	−46.95	−46.19 (−48.18, −44.19)	0.2
NEFA AUC 5h (mmol/L)	Saline	−479.92	−314.04	165.88 (144.42, 187.34)	0.57
	Intralipid	−176.11	−1688.26	−1512.15 (−1596.83, −1427.48)	0.31

When the effect of exercise on intralipid-induced insulin resistance was analyzed between the PCOS and control group, no significant differences were found (Table 7).

Analysis of testosterone and SHBG before and after exercise did not differ (data not shown).

Given the importance that obesity may have on insulin sensitivity, subjects with a BMI >30 were excluded from a subanalysis of 7 PCOS and 9 control subjects, but the M value did not differ significantly from the results above (data not shown).

## Discussion

It is well recognized that women with PCOS are more IR than their weight and age matched controls (26). In the hyperinsulinaemic euglycaemic clamp the rate of glucose infused is equal to the rate of whole-body glucose disposal or metabolizable glucose (M value) and reflects the amount of exogenous glucose necessary to fully compensate for the hyperinsulinemia; in insulin resistance this M value is lower and this was reflected in the lower values seen for the PCOS patients compared to controls in the saline infusion and particularly in the lipid infusion that exacerbated the insulin resistance. The tolerance to an acute rise in NEFA in PCOS has not been studied in comparison with non-PCOS women. Before exercise, an acute rise in NEFA with the lipid infusion lowered the rate of insulin stimulated glucose disposal in skeletal muscle (i.e., increased insulin resistance) by a greater magnitude in PCOS than age and BMI matched controls. This could be related to their underlying insulin signaling defect or to their lower VO_2_ max and higher waist circumference when compared with controls. This also suggests that the underlying dyslipidemia found in PCOS may be a major contributor to the exacerbation of the underlying IR, and may be reflected in the improvement in IR with anti-lipid statin therapy (27).

Ectopic fat in muscle, liver and beta cells (2) through triglyceride storage leads to a decreased rate of mitochondrial lipid oxidation in skeletal muscle, resulting in accumulation of intra-myocellular lipid metabolites such as the long chain fatty acids Acyl Co A, diacylglycerol (DAG) and ceramides (5, 6). These are thought to interfere with insulin signaling mediated glucose transport, and thus result in IR in skeletal muscle (7, 8); therefore, if this is the fundamental cause of IR in PCOS, exercise, through a reduction in intra-myocellular fat metabolites, would improve IR relative to that of the control subjects (11). At the end of the 8 week moderate intensity exercise, women with PCOS improved fasting HOMA-IR and insulin stimulated glucose disposal in skeletal muscle with a significant improvement in physical fitness similar to controls. PCOS patients became more tolerant of lipid induced IR after exercise and became comparable with controls prior to exercise, but this was not reversed to that level seen for normal controls following exercise. This improvement is likely due to increased fat oxidation with moderate intensity exercise (12), and a reduction in intra-myocellular fat metabolites would improve IR (11). In addition, moderate intensity exercise has been proven to improve insulin sensitivity in conjunction with favorable alterations in lipid partitioning and an enhanced lipid oxidative capacity within muscle in healthy adults (28). Though previous studies have shown the benefit of exercise and weight loss with a reduction of IR in PCOS (29, 30) and a reduction in muscle lipid (31), this is the first time a study has shown that endurance exercise attenuates lipid induced IR. This suggests that PCOS women who undertake moderate intensity exercise for 180 min per week could tolerate an acute fat load comparable to the level observed in controls and possibly reduce the risk of adverse metabolic outcomes. The absolute increase in the dynamic IR induced by the lipid infusion did not differ before and after exercise for both the controls and PCOS subjects, indicating that the underlying mechanism of the IR was unchanged by exercise. However, IR could be modified with exercise in the PCOS insulin resistant individuals, but could not be improved upon if insulin resistance was already normal. PCOS subjects who did the exercise had a response i.e. the percent reduction in glucose disposal rate to the lipid infusion was similar to healthy subjects with no regular exercise.

Following exercise, there was a significant reduction in NEFA AUC during the lipid saline infusion for controls and for both the saline and lipid infusions for the PCOS group, suggesting exercise induced effective removal of circulating NEFA (32). TG levels fell with exercise, thus it is unlikely to be due to an increased uptake of NEFA by the liver to synthesize very low density lipoproteins (VLDL). Therefore, the fall in NEFA levels after exercise in this study may well indicate increased uptake of NEFA by skeletal muscle in PCOS.

PCOS has a profound effect on peripheral insulin resistance due to a reduction in insulin receptor substrate-1 (IRS-1) associated phosphatidylinositol (PI) 3-kinase activity in skeletal muscle (33). This underlying intrinsic defect in the proximal insulin signaling pathway could explain their lipid intolerance. However, a previous study showed that intralipid had no effect on proximal signaling (34). Therefore, it is more likely that acutely elevated NEFA levels act synergistically rather than enhancing the underlying insulin signaling defect in skeletal muscle glucose transport in PCOS. A recent study showed that endurance-trained athletes with high mitochondrial oxidative capacity had lower lipid induced IR than untrained subjects (35). This might explain why PCOS subjects with less physical fitness had more severe lipid induced IR than controls before exercise. Analysis of testosterone and SHBG before and after the 8 week exercise showed no change, in accord with others (36).

Abdominal obesity is associated with increased delivery of NEFA to non- adipose tissue, either due to enhanced mobilization of NEFA from adipose tissue due to decreased effective inhibition of insulin on hormone sensitive lipase (37) or to decreased entrapment of NEFA by adipose tissue during the postprandial period (38). It has been recognized that elevated NEFA interferes with skeletal muscle glucose transport via increased production of intermediate lipid metabolites (39). Corbould et al. reported that an intrinsic insulin signaling defect only inhibited insulin mediated glucose transport in the presence of lipidemia in skeletal muscle of PCOS in *in vitro* studies (18, 40). The present *in vivo* study confirms the importance of high NEFA levels increasing skeletal muscle IR in PCOS. Therefore, IR will be enhanced in PCOS in those with a high fat diet, or acute weight gain with high NEFA availability. The M value did not differ significantly when subjects with a BMI >30 were excluded, but this is likely due to the observation that insulin resistance may decrease 15 ± 8% with BMI (25) and the sample size was too small to detect this.

Bruce et al. investigated the effect of 8 weeks of moderate intensity endurance exercise on the rate of mitochondrial fatty acid oxidation and lipid content in muscle of obese subjects and their relation to glucose tolerance. It was found that endurance exercise improved glucose tolerance with an increase in mitochondrial fatty acid oxidation (11). A recent study reported that trained individuals were more tolerant to lipid induced insulin resistance than untrained subjects because they had higher mitochondrial oxidative capacities in skeletal muscle (35). Therefore, adequate mitochondrial lipid oxidation is required to maintain the balance of fatty acid uptake and utilization to protect excessive accumulation of intra-myocellular triglycerides and their metabolites. In women with PCOS, the impaired insulin-stimulated total oxidative and non-oxidative glucose disposal are associated with a consistent downregulation of mitochondrial oxidative phosphorylation gene expression (OXPHOS) in skeletal muscle that couples with reduced levels of peroxisome proliferator-activated receptor gamma co-activator alpha (PGC-1alpha) (41). Hence, this defect could also be a plausible explanation for the failure of exercise to reverse completely lipid-induced insulin resistance in PCOS in this study.

This failure of complete reversal of lipid induced insulin resistance by exercise was also observed in healthy subjects in this study. Therefore, it may well be due to the time gap between the lipid infusion test and exercise activity. In other words, performing the lipid trial immediately after the exercise may have given a different result, particularly if the effect of exercise was not sustained. This had been seen in a previous study in which an hour of prior leg exercise before lipid infusion alleviated the lipid induced insulin resistance in healthy subjects (42).

The strength of this study was using gold standard methodology for this intensive interventional study and the well supervised exercise intervention. However, the limitations were that this was a small group of women with PCOS, though all fulfilled all three of the diagnostic criteria for its diagnosis thereby reducing heterogeneity; the groups were not well matched for BMI and age. The diagnostic criteria to define the PCOS group would likely not have altered the results here as IS was reported to be no different between NIH and Rotterdam criteria (15).

In summary, insulin sensitivity improved with exercise in the PCOS group alone showing that IR can be modified, though likely transiently. However, the maximal IR response to the lipid infusion did not differ within and between control and PCOS subjects indicating that the fundamental mechanism underlying insulin resistance was unchanged with exercise.

## Ethics statement

The study was carried out in accordance with the recommendations of the International Council for Harmonization Guidelines for Good Clinical Practice (ICH GCP) with written informed consent from all subjects. All subjects gave written informed consent in Accordance with the Declaration of Helsinki. The protocol was approved by the Humber Bridge Regional Ethics Committee.

## Author contributions

MA assisted with study design, performed the study, and assisted with manuscript preparation. SA and DS were responsible for overall study design and manuscript preparation. AR performed the statistics. AB, EK, RK, and RV assisted with the study and manuscript preparation.

### Conflict of interest statement

The authors declare that the research was conducted in the absence of any commercial or financial relationships that could be construed as a potential conflict of interest.

## References

[1] Boden G. Role of fatty acids in the pathogenesis of insulin resistance and NIDDM. Diabetes (1997) 46:3–10. 10.2337/diab.46.1.3 8971073

[2] McQuaid SE Hodson L Neville MJ Dennis AL Cheeseman J Humphreys SM. Downregulation of adipose tissue fatty acid trafficking in obesity: a driver for ectopic fat deposition? Diabetes (2011) 60:47–55. 10.2337/db10-0867 20943748 PMC3012196

[3] UK Hypoglycaemia Study Group Risk of hypoglycaemia in types 1 and 2 diabetes: effects of treatment modalities and their duration. Diabetologia (2007) 50:1140–7. 10.1007/s00125-007-0599-y 17415551

[4] Weiss R Dufour S Taksali SE Tamborlane WV Petersen KF Bonadonna RC. Prediabetes in obese youth: a syndrome of impaired glucose tolerance, severe insulin resistance, and altered myocellular and abdominal fat partitioning. Lancet (2003) 362:951–7. 10.1016/S0140-6736(03)14364-4 14511928 PMC2995523

[5] Boden G. Fatty acid-induced inflammation and insulin resistance in skeletal muscle and liver. Curr Diab Rep. (2006) 6:177–81. 10.1007/s11892-006-0031-x 16898568

[6] Boden G Lebed B Schatz M Homko C Lemieux S. Effects of acute changes of plasma free fatty acids on intramyocellular fat content and insulin resistance in healthy subjects. Diabetes (2001) 50:1612–7. 10.2337/diabetes.50.7.1612 11423483

[7] Ellis BA Poynten A Lowy AJ Furler SM Chisholm DJ Kraegen EW. Long-chain acyl-CoA esters as indicators of lipid metabolism and insulin sensitivity in rat and human muscle. Am J Physiol Endocrinol Metab. (2000) 279:E554–60. 10.1152/ajpendo.2000.279.3.E554 10950822

[8] Itani SI Ruderman NB Schmieder F Boden G. Lipid-induced insulin resistance in human muscle is associated with changes in diacylglycerol, protein kinase C, and IkappaB-alpha. Diabetes (2002) 51:2005–11. 10.2337/diabetes.51.7.2005 12086926

[9] Baldeweg SE Golay A Natali A Balkau B Del Prato S Coppack SW. Insulin resistance, lipid and fatty acid concentrations in 867 healthy Europeans. European Group for the Study of Insulin Resistance (EGIR). Eur J Clin Invest. (2000) 30:45–52. 10620001 10.1046/j.1365-2362.2000.00597.x

[10] Boden G Jadali F White J Liang Y Mozzoli M Chen X. Effects of fat on insulin-stimulated carbohydrate metabolism in normal men. J Clin Invest. (1991) 88:960–6. 10.1172/JCI115399 1885781 PMC295496

[11] Bruce CR Thrush AB Mertz VA Bezaire V Chabowski A Heigenhauser GJ. Endurance training in obese humans improves glucose tolerance and mitochondrial fatty acid oxidation and alters muscle lipid content. Am J Physiol Endocrinol Metab. (2006) 291:E99–107. 10.1152/ajpendo.00587.2005 16464906

[12] Romijn JA Coyle EF Sidossis LS Gastaldelli A Horowitz JF Endert E. Regulation of endogenous fat and carbohydrate metabolism in relation to exercise intensity and duration. Am J Physiol. (1993) 265(3 Pt 1):E380–91. 10.1152/ajpendo.1993.265.3.E380 8214047

[13] Schenk S Horowitz JF. Acute exercise increases triglyceride synthesis in skeletal muscle and prevents fatty acid-induced insulin resistance. J Clin Invest. (2007) 117:1690–8. 10.1172/JCI30566 17510709 PMC1866251

[14] Stepto NK Cassar S Joham AE Hutchison SK Harrison CL Goldstein RF. Women with polycystic ovary syndrome have intrinsic insulin resistance on euglycaemic-hyperinsulaemic clamp. Hum Reprod. (2013) 28:777–84. 10.1093/humrep/des463 23315061

[15] Cassar S Misso ML Hopkins WG Shaw CS Teede HJ Stepto NK. Insulin resistance in polycystic ovary syndrome: a systematic review and meta-analysis of euglycaemic-hyperinsulinaemic clamp studies. Hum Reprod. (2016) 31:2619–31. 10.1093/humrep/dew243 27907900

[16] Teede HJ Misso ML Costello MF Dokras A Laven J Moran L. Recommendations from the international evidence-based guideline for the assessment and management of polycystic ovary syndrome. Hum Reprod. (2018) 110:364–79. 10.1016/j.fertnstert.2018.05.004 30033227 PMC6939856

[17] Corbould A Kim YB Youngren JF Pender C Kahn BB Lee A. Insulin resistance in the skeletal muscle of women with PCOS involves intrinsic and acquired defects in insulin signaling. Am J Physiol Endocrinol Metab. (2005) 288:E1047–54. 10.1152/ajpendo.00361.2004 15613682

[18] Diamanti-Kandarakis E Dunaif A. Insulin resistance and the polycystic ovary syndrome revisited: an update on mechanisms and implications. Endocr Rev. (2012) 33:981–1030. 10.1210/er.2011-1034 23065822 PMC5393155

[19] Group REA-SPCW Revised 2003 consensus on diagnostic criteria and long-term health risks related to polycystic ovary syndrome. Fertil Steril. (2004) 81:19–25 10.1016/j.fertnstert.2003.10.004 14711538

[20] Zuniga-Guajardo S Jimenez J Angel A Zinman B. Effects of massive obesity on insulin sensitivity and insulin clearance and the metabolic response to insulin as assessed by the euglycemic clamp technique. Metab Clin Exp. (1986) 35:278–82. 10.1016/0026-0495(86)90214-3 3512959

[21] DeFronzo RA Tobin JD Andres R. Glucose clamp technique: a method for quantifying insulin secretion and resistance. Am J Physiol. (1979) 237:E214–23. 382871 10.1152/ajpendo.1979.237.3.E214

[22] Kuipers H Verstappen FT Keizer HA Geurten P van Kranenburg G. Variability of aerobic performance in the laboratory and its physiologic correlates. Int J Sports Med. (1985) 6:197–201. 10.1055/s-2008-1025839 4044103

[23] Friedewald WT Levy RI Fredrickson DS. Estimation of the concentration of low-density lipoprotein cholesterol in plasma, without use of the preparative ultracentrifuge. Clin Chem. (1972) 18:499–502. 4337382

[24] Matthews DR Hosker JP Rudenski AS Naylor BA Treacher DF Turner RC. Homeostasis model assessment: insulin resistance and beta-cell function from fasting plasma glucose and insulin concentrations in man. Diabetologia (1985) 28:412–9. 3899825 10.1007/BF00280883

[25] Matthews JN Altman DG Campbell MJ Royston P. Analysis of serial measurements in medical research. BMJ (1990) 300:230. 10.1136/bmj.300.6719.230 2106931 PMC1662068

[26] Ehrmann DA. Insulin resistance and polycystic ovary syndrome. Curr Diab Rep. (2002) 2:71–6. 12643125 10.1007/s11892-002-0061-y

[27] Sathyapalan T Kilpatrick ES Coady A-M Atkin SL Te Effect of atorvastatin in patients with polycystic ovary syndrome: a randomized double-blind placebo-controlled study J Clin Endocrinol Metab. (2009) 94:103–8. 10.1210/jc.2008-1750 18940877

[28] Dube JJ Amati F Stefanovic-Racic M Toledo FG Sauers SE Goodpaster BH. Exercise-induced alterations in intramyocellular lipids and insulin resistance: the athlete's paradox revisited. (2008) 294:E882–8. 10.1152/ajpendo.00769.2007 18319352 PMC3804891

[29] Thomson RL Buckley JD Noakes M Clifton PM Norman RJ Brinkworth GD. The effect of a hypocaloric diet with and without exercise training on body composition, cardiometabolic risk profile, and reproductive function in overweight and obese women with polycystic ovary syndrome. Clin Endocrinol Metab. (2008) 93:3373–80. 10.1210/jc.2008-0751 18583464

[30] Bruner B Chad K Chizen D. Effects of exercise and nutritional counseling in women with polycystic ovary syndrome. Appl Physiol Nutr Metab. (2006) 31:384–91. 10.1139/h06-007 16900227

[31] Hutchison SK Teede HJ Rachon D Harrison CL Strauss BJ Stepto NK. Effect of exercise training on insulin sensitivity, mitochondria and computed tomography muscle attenuation in overweight women with and without polycystic ovary syndrome. Diabetologia (2012) 55:1424–34. 10.1007/s00125-011-2442-8 22246378

[32] van Hall G. The physiological regulation of skeletal muscle fatty acid supply and oxidation during moderate-intensity exercise. Sports Med. (2015) 45(Suppl 1):S23–32. 10.1007/s40279-015-0394-8 26553490 PMC4672010

[33] Dunaif A Wu X Lee A Diamanti-Kandarakis E. Defects in insulin receptor signaling *in vivo* in the polycystic ovary syndrome (PCOS). Am J Physiol Endocrinol Metab. (2001) 281:E392–9. 10.1152/ajpendo.2001.281.2.E392 11440917

[34] Storgaard H Jensen CB Bjornholm M Song XM Madsbad S Zierath JR. Dissociation between fat-induced *in vivo* insulin resistance and proximal insulin signaling in skeletal muscle in men at risk for type 2 diabetes. J Clin Endocrinol Metab. (2004) 89:1301–11. 10.1210/jc.2003-031243 15001626

[35] Phielix E Meex R Ouwens DM Sparks L Hoeks J Schaart G. High oxidative capacity due to chronic exercise training attenuates lipid-induced insulin resistance. Diabetes (2012) 61:2472–8. 10.2337/db11-1832 22787138 PMC3447923

[36] Almenning I Rieber-Mohn A Lundgren KM Shetelig Lovvik T Garnaes KK Moholdt T. Effects of high intensity interval training and strength training on metabolic, cardiovascular and hormonal outcomes in women with polycystic ovary syndrome: a pilot study. PLoS ONE (2015) 10:e0138793. 10.1371/journal.pone.0138793 26406234 PMC4583183

[37] Coppack SW Evans RD Fisher RM Frayn KN Gibbons GF Humphreys SM. Adipose tissue metabolism in obesity: lipase action *in vivo* before and after a mixed meal. Metab Clin Exp. (1992) 41:264–72. 10.1016/0026-0495(92)90269-G 1542265

[38] Fielding BA Callow J Owen RM Samra JS Matthews DR Frayn KN. Postprandial lipemia: the origin of an early peak studied by specific dietary fatty acid intake during sequential meals. Am J Clin Nutr. (1996) 63:36–41. 8604667 10.1093/ajcn/63.1.36

[39] Yu C Chen Y Cline GW Zhang D Zong H Wang Y. Mechanism by which fatty acids inhibit insulin activation of insulin receptor substrate-1 (IRS-1)-associated phosphatidylinositol 3-kinase activity in muscle. J Biol Chem. (2002) 277:50230–6. 10.1074/jbc.M200958200 12006582

[40] Corbould A Dunaif A The adipose cell lineage is not intrinsically insulin resistant in polycystic ovary syndrome. Metab Clin Exp. (2007) 56:716–22. 10.1016/j.metabol.2006.12.021 17445549 PMC2427369

[41] Skov V Glintborg D Knudsen S Jensen T Kruse TA Tan Q. Reduced expression of nuclear-encoded genes involved in mitochondrial oxidative metabolism in skeletal muscle of insulin-resistant women with polycystic ovary syndrome. Diabetes (2007) 56:2349–55. 10.2337/db07-0275 17563058

[42] Pehmoller C Brandt N Birk JB Hoeg LD Sjoberg KA Goodyear LJ. Exercise Alleviates lipid-induced insulin resistance in human skeletal muscle-signaling interaction at the level of TBC1 domain family member 4. Diabetes (2012) 61:2743–52. 10.2337/db11-1572 22851577 PMC3478539

